# Covalently tethered transforming growth factor beta in PEG hydrogels promotes chondrogenic differentiation of encapsulated human mesenchymal stem cells

**DOI:** 10.1007/s13346-012-0090-2

**Published:** 2012-09-18

**Authors:** Joshua D. McCall, Jacob E. Luoma, Kristi S. Anseth

**Affiliations:** 1 Department of Chemical and Biological Engineering, University of Colorado at Boulder, Boulder, CO USA; 2 Howard Hughes Medical Institute, Chevy Chase, MD USA; 3 University of Colorado at Boulder, Campus Box 596, Boulder, CO 80309 USA

**Keywords:** Immobilized growth factors, Hydrogels, Chondrogenesis, Stem cell differentiation, Photopolymerization, Cell scaffold

## Abstract

Methods to precisely control growth factor presentation in a local and sustained fashion are of increasing interest for a number of complex tissue engineering applications. The cytokine transforming growth factor beta (TGF*β*) plays a key role in promoting the chondrogenic differentiation of human mesenchymal stem cells (hMSCs). Traditional chondrogenic approaches utilize soluble delivery, an approach with limited application for clinical translation. In this work, we introduce a reactive thiol onto TGF*β* and covalently tether the growth factor into poly(ethylene glycol) (PEG) hydrogels using a photoinitiated thiol-acrylate polymerization mechanism. We demonstrate the bioactivity of thiolated TGF*β*, before and after polymerization, using a SMAD2 reporter cell line. hMSCs were encapsulated in PEG hydrogels with and without tethered TGF*β*, and subsequently assayed for glycosaminoglycan and collagen II production as indicators of chondrogenesis. Over a 21-day time course, tethered TGF*β* promoted chondrogenesis at levels similar to a positive control using solubly dosed growth factor. These results provide evidence that tethered TGF*β* materials can be successfully used to promote chondrogenic differentiation of MSCs.

## Introduction

There is growing interest in methods to sequester and present bioactive therapeutic proteins to cells immobilized in 3D matrices, specifically for application in the areas of stem cell culture and regenerative medicine [1]. Numerous biological processes are regulated through protein signaling. For example, cytokines and chemokines are a particularly attractive target for tissue engineering applications, since these biomolecules are directly involved in controlling critical cell functions like proliferation, differentiation, and chemotaxis. Typically, these proteins are potent at concentrations as low as nano- to picomolar [2–4], and many of them function via interaction with extracellular surface receptors [5], which then regulate changes in gene expression. Growth factors are commonly dosed solubly via culture media in vitro; however, in vivo, these proteins are sequestered in the extracellular matrix, and these sequestered factors are then available to nearby cells.

Hydrogel scaffolds are increasingly utilized to reconstruct and study such complex 3D interactions. The cellular microenvironment plays a key role in providing diverse cues that direct cell function in vivo [6, 7]. The extracellular matrix itself provides not only a niche for cell attachment, but also acts as a storage depot for signaling proteins [2, 6, 7], and there is growing interest in strategies that recapitulate aspects of these functionalities using synthetic hydrogel scaffolds. Sequestered protein approaches also address practical implications for growth factor delivery. Growth factors are typically cross-reactive with multiple cell types and are reported to have short serum half-lives [3], limitations that often necessitate localized presentation, rather than systemic dosing. Additionally, immobilizing such proteins allows control of total dose delivered and can increase the safety and persistence of signaling. Indeed, immobilizing growth factors in a bioactive, physiologically relevant context is an important step towards clinical implementation for regenerative medicine as a whole. Recently, a number of growth factors have been immobilized into synthetic substrates, including VEGF [8], PDGF [9], and transforming growth factor beta (TGF*β*) [10–13] and shown to maintain bioactivity while tethered. Covalent incorporation of such potent cell-directing proteins into hydrogels suggests an attractive approach to design cell delivery scaffolds for locally directing and guiding cell properties important for tissue regeneration.

In this regard, we were particularly interested in modifying poly(ethylene glycol) (PEG) hydrogels with proteins, as these systems are broadly explored for cell delivery applications [14–18] and the low fouling properties of PEG render it useful for examining the effects of particular protein signals on encapsulated cell function. One widely used approach for forming PEG hydrogels is through the photoinitiated chain polymerization of di(meth)acrylated PEG monomers. Photopolymerization allows for precise spatial and temporal control of polymerization and can be carried out at physiological temperature in aqueous conditions. The resulting crosslinked PEG hydrogels have enjoyed wide utilization for encapsulation of numerous cells types with high cell survival reported following photoencapsulation [19–21]. However, less explored is the mixed-mode thiol-acrylate photopolymerization [15] that provides a facile method for incorporating thiol-functionalized molecules during polymerization of acrylate functional groups [22]. Our group has successfully used this approach to incorporate a number of peptide functionalities into PEG hydrogels, including adhesive motifs [22, 23], affinity-binding ligands [24, 25], and enzyme-cleavable peptides [22, 26]; however, proteins have been less explored. We hypothesized that a thiolated growth factor, such as TGF*β*, could be incorporated into PEG hydrogels via a thiol-acrylate photopolymerization while maintaining its activity and accessibility for cell binding. Specifically, we were interested in exploring how this method might be useful for locally promoting the chondrogenesis of human mesenchymal stem cells (hMSCs) in the absence of exogenously delivered TGF*β*.

**Scheme 1:**
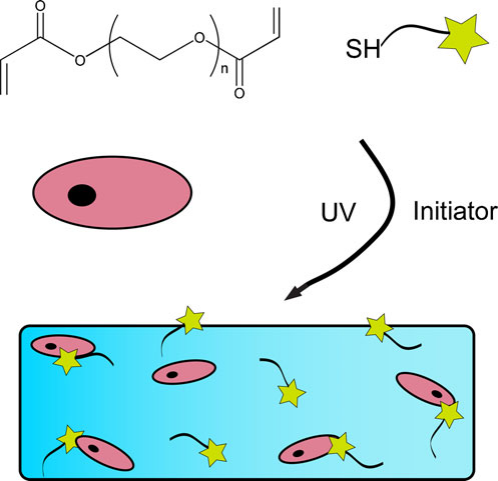
Photoinitiated thiol-acrylate polymerization scheme

In this work, TGF*β* was thiolated and covalently incorporated into PEG diacrylate hydrogels using a photoinitiated thiol-acrylate polymerization scheme (Scheme 1). We confirmed that thiolated TGF*β* had bioactivity similar to that of the native, unmodified growth factor, using a cell reporter assay for SMAD2 signaling. By varying the concentration of thiolated TGF*β* in the monomer solution, the total growth factor concentration in the final gel was readily controlled, as determined by a modified surface ELISA. Bioactivity of the tethered growth factor and the ability for it to signal encapsulated cells was further confirmed using the SMAD2 reporter cell line. Finally, we demonstrate the potential of this material platform by encapsulating hMSCs in order to promote chondrogenic differentiation. mesenchymal stem cells (MSCs) in tethered TGF*β* hydrogels produced extracellular matrix proteins (ECM) proteins indicative of chondrogenic differentiation. Namely, MSC cultured in gels with tethered TGF*β* produced glycosaminoglycan (GAG) and collagen II at levels similar to or exceeding that of positive controls, where TGF*β* was dosed solubly in the culture media. We believe these results indicate the clinical potential of tethered growth factor biomaterial platforms as a cell delivery system for tissue engineering applications demanding tunable control of bioactive protein signals in a local and sustained manner.

## Materials and methods

### Cell culture and expansion

hMSCs were isolated from bone marrow aspirate (Lonza Biosciences) as detailed elsewhere [27]. Cells were cultured in growth medium consisting of low-glucose DMEM supplemented with 10 % fetal bovine serum, 1 μg/mL amphotericin B, 50 U/mL penicillin, 50 μg/mL streptomycin, and 20 μg/mL gentamicin. Cultures were maintained at 5 % CO_2_ and 37 °C, and hMSCs were passaged up to three times prior to encapsulation studies.

PE25 cells were cultured in growth medium identical to that used for hMSCs. For analysis of TGF*β* bioactivity, plated cells were cultured in serum-free DMEM supplemented with 1 μg/mL amphotericin B, 50 U/mL penicillin, 50 μg/mL streptomycin, and 20 μg/mL gentamicin, augmented with either native (non-thiolated) or thiolated TGF*β* at 5 ng/mL.

### Monomer synthesis

Poly(ethylene glycol) (*M*_n_ = 4,600) diacrylate (PEGDA) was synthesized as previously described [25]. Briefly, poly(ethylene glycol) (PEG) was dissolved in toluene and reacted with acryoyl chloride in the presence of triethylamine overnight under argon. The product was filtered through alumina oxide and neutralized with sodium carbonate. The filtrate was then precipitated in cold ethyl ether. Proton NMR was used to verify yield; typical functionalization for the PEG material used in this study was greater than 95 %.

### Growth factor incorporation into PEG hydrogels

2-Iminothiolane (Pierce) was purchased and used to thiolate TGF*β* (isoform 1) (Peprotech) according to the manufacturer's recommendations. Following the reaction, thiolated-TGF*β* was aliquoted until used. Thiolated-TGF*β* was then reacted into PEG hydrogels via thiol-acrylate polymerization with the PEGDA monomer at concentrations of 0, 1, 10, or 100 nM. The hydrogels with the tethered growth factor were incubated overnight in phosphate-buffered saline (PBS), and the TGF*β* surface density was measured using a modified ELISA as described elsewhere [12].

Briefly, gels were incubated overnight in a blocking solution of 1 % bovine serum albumin (BSA) in PBS containing 0.05 % Tween-20 (PBS-T). Gels were washed 3× in PBS-T prior to incubation with a biotinylated anti-TGF*β* antibody (eBioscience) for 1 h at room temperature. Gels were washed 3x in PBS-T, and incubated with avidin-HRP (eBioscience) for 30 min, and washed 3× in PBS-T. After washing overnight in PBS-T, gels were incubated with 3,3′,5,5′ tetramethylbenzidine substrate until color developed, at which time the reaction was stopped using 2 N sulfuric acid. The solutions were then measured for optical density at 450 nm using a Bioteck H1 spectrophotometer.

### Bioactivity of thiolated TGF*β*

PE25 cells were plated in 12-well plates at a density of 200,000 cells/well, then incubated with serum-free DMEM media augmented with 1 ng/mL native (non-thiolated) TGF*β* (Peprotech) or thiolated TGF*β*. Cells were incubated overnight at 37 °C, then analyzed using Glo–Lysis components from Promega.

Alternatively, PE25 cells were encapsulated in PEG gels using a formulation of 10 wt% PEGDA, 1 mM lithium acyl phosphinate (LAP) initiator, 1 mM Cys–Arg–Gly–Asp–Ser (CRGDS) peptide, and 0, 12.5 nM, 25 nM, or 50 nM thiolated-TGF*β*. PE25 cells were suspended at densities of 1, 5, 10, and 25 million cells/mL of solution, and cell-laden hydrogels were formed at a volume of 40 μL (O.D. ~5 mm, thickness ~2 mm) using photoinitiation (*I*_o_ ~3.5 mW/cm^2^ at *λ* = 365 nm) for 180 s. Immediately following encapsulation, hydrogels were placed into growth medium in 48-well plates and incubated overnight at 37 °C, 5 % CO_2_. Following incubation, hydrogels were placed in Glo–Lysis buffer (Promega) for not less than 10 min and frozen at −70 °C for greater than 2 h. Lysate was incubated with luciferase substrate (Promega) and luminescence was monitored on a Biotek H1 Hybrid spectrophotometer.

### hMSC encapsulation in PEGDA hydrogels

hMSCs were encapsulated at 10 × 10^6^ cells/mL in 10-wt% PEGDA (*M*_n_ = 4,600) solution, with 2 mM CRGDS adhesive peptide and 1 mM LAP photoiniator. Thiolated TGF*β* concentrations of 0, 1, 10, or 100 nM were used to form tethered hydrogels. Polymerization was initiated with light (*I*_o_ = ~3.5 mW/cm^2^, *λ* = 365 nm) under sterile conditions to create 40 μL gels (O.D. ~5 mm, thickness ~2 mm), and cell-laden gels were immediately placed into experimental culture medium. Growth medium (described previously) was used for a negative control, and chondrogenic medium (high-glucose DMEM, 1 μg/mL amphotericin B, 50 U/mL penicillin, 50 μg/mL streptomycin, 20 μg/mL gentamicin, 100 nM dexamethasone, ITS + premix (6.25 μg/mL bovine insulin, 6.25 μg/mL transferrin, 6.25 μg/mL selenous acid, 5.33 μg/mL linoleic acid, and 1.25 μg/mL bovine serum albumin), 100 μg/mL sodium pyruvate, and 50 μg/mL ascorbic acid 2-phosphate) with 5 ng/mL soluble TGF*β* was used as a positive control for chondrogenesis. Hydrogels with tethered TGF*β* (1, 10, or 100 nM) were cultured in chondrogenic medium, but in the absence of soluble TGF*β* added to the media. Samples were harvested at days 0, 7, 14, and 21 for analysis of chondrogenesis.

### DNA quantification of encapsulated hMSCs

Immediately following photoencapsulation, cell-laden hydrogels (*n* = 3) were placed into an enzymatic digest buffer (125 μg/mL papain (Worthington Biochemical), 10 mM cysteine) overnight at 60 °C, then frozen prior to analysis. Samples were similarly harvested at days 7 and 14, and these solutions were then assayed for DNA content using a Picogreen assay (Invitrogen) to quantify changes in cell number between various culture conditions.

### Glycosaminoglycan production assay

Dimethyl methylene blue (DMMB) assay was used to quantify GAG production in hydrogel scaffolds. Cell-laden gels were harvested at pre-determined timepoints (days 0, 7, and 14) and incubated overnight at 60 °C in 125 μg/mL papain, 10 mM cysteine. Samples were then incubated with DMMB dye and analyzed for chondroitin sulfate content by measuring absorbance at 525 nm using a Biotek H1 spectrophotometer and comparing to a standard series for quantification.

### Histological analyses

Samples were harvested after 20 days of culture and fixed in 4 % formalin, 30 % sucrose at 4 °C for 48 h. Following fixing, gels were frozen and cryosectioned at 30 μm using a Leica CM1850 Cryostat. Samples were stained for Safranin-O on a Leica Autostainer XL and imaged in bright field (×40) on a Nikon inverted microscope. For immunostaining, sections were blocked in 10 % goat serum with 1 % BSA, then incubated with antibodies for collagen type II (abcam) and CD105 (Sigma) for 2 h. Alexa Fluor-conjugated secondary antibodies (Invitrogen) and 4′,6-diamidino-2-phenylindole (DAPI) were used to fluorescently label proteins of interest. Images were collected on a Zeiss LSM710 scanning confocal microscope. Quantification of collagen II or CD-105-positive cells was performed on ×10 images (three per sample, *n* = 3 samples) by counting the number of cells positive for either collagen II or CD-105, then normalizing to total cell nuclei using DAPI.

### Statistical analysis

All data were plotted and analyzed using Graphpad Prism 5.0 software. Error bars are plotted as the standard deviation for three replicate conditions, unless otherwise noted. Statistical differences were calculated using a Student's *t* test.

## Results and discussion

### Activity of thiolated TGF*β* in PEG hydrogels

**Fig. 1:**
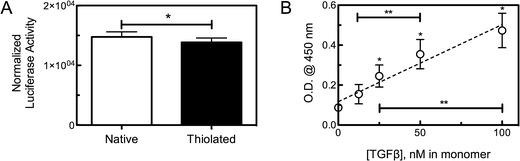
TGF*β* can be covalently incorporated into PEG hydrogels. **a** Results from a PE25 cell assay show that thiolated TGF*β* provides identical bioactivity to that of native (non-thiolated) growth factor. **b** Surface ELISA results show detection of tethered TGF*β* in PEG hydrogels, up to a concentration of 100 nM. Monomer solutions containing 0, 12.5, 25, 50, or 100 nM thiolated TGF*β* were photopolymerized (*I*_o_ = 3.5 mW/cm^2^, *λ* = 365 nm) and allowed to equilibrate in 1 % BSA. A surface ELISA showed a linear correlation between detectable TGF*β* and its concentration in the initial monomer solution, providing a facile method to control the total protein payload. *p < 0.05, denotes values statistically non-zero, and **p < 0.05, denotes statistical differences between concentrations. Both data sets are presented as mean ± s.d. (*n* = 5)

To introduce a functional group on native TGF*β* for subsequent conjugation to PEG, the protein was thiolated via the reaction of 2-iminothiolane with primary amines, such as that found on the N-terminus. During photopolymerization of PEG hydrogels, this thiol can be covalently tethered into the network via chain transfer from a propagating radical on a growing polyacrylate kinetic chain, creating a pendant protein presentation [28, 29]. For confirmation of TGF*β* activity following thiolation, a PE25 cell assay was utilized. This line has been permanently transfected with a luciferase reporter for SMAD2 activation, an indicator of TGF*β* bioactivity [30]. PE25 cells were plated and incubated in medium containing either native (non-thiolated) or thiolated TGF*β* at identical concentrations. Following an incubation period of 18 h, the cells were lysed and assayed for luciferase activity, which represents relative TGF*β* bioactivity (Fig. 1a). Results indicate that thiolation with 2-iminothiolane did not significantly decrease the bioavailability of TGF*β* (*p* < 0.005).

Thiolated TGF*β* was then added to a PEGDA (*M*_n_ = 4,600) monomer (10 wt %) solution at 0, 12.5, 25, 50, or 100 nM, and the solutions were irradiated (*I*_o_ ~ 3.5 mW/cm^2^, *λ* = 365 nm) for 180 s. The resulting hydrogels were then assayed for detectable TGF*β* concentration using a modified surface ELISA [12]; results are presented in Fig. 1b. The ELISA confirms TGF*β* incorporation into the gel, and results show a linear response for TGF*β* in the 0–100-nM concentration range. However, as this method only utilizes antibody recognition, further confirmation of bioactivity of the tethered TGF*β* in the PEG hydrogel platform was required, using an encapsulation study with the PE25 reporter cell line.

### Tethered TGF*β* bioactivity in 3D culture

**Fig. 2:**
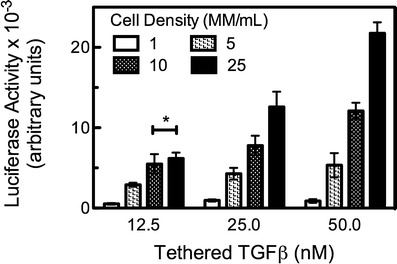
Tethered TGF*β* hydrogels present a bioactive signal to encapsulated cells. PE25 cells were encapsulated into hydrogels formed with 12.5-, 25-, or 50-nM tethered TGF*β*. Cells were encapsulated at densities of 1, 5, 10, or 25 million cells/mL. For all TGF*β* concentrations tested, increasing the cell density from 1 to 10 MM cells/mL produced increased luciferase activity, a measure of the bioactive TGF*β* concentration detected by the cells. Hydrogels with 12.5-nM tethered TGF*β* had no statistically significant increase in luciferase activity between 10 and 25 MM cells/mL, indicating that the amount of available tethered growth factor is limiting. At higher TGF*β* concentrations (25 and 50 nM), luciferase activity increased with increasing cell number. For a given TGF*β* concentration, *t* test showed significant difference between all luciferase values except those indicated with *asterisk*. Results are presented as mean activity ± s.d. (*n* = 5)

After confirming the tunability of TGF*β* concentration in PEG hydrogels, we next wanted to investigate the bioactivity of TGF*β* tethered in a 3D culture system using the thiol-acrylate polymerization reaction. PEG diacrylate monomer (10-wt%) was formulated with varying concentrations of TGF*β*: 0, 12.5, 25, and 50 nM. Concurrently, the seeding density of PE25 cells was varied, with cells encapsulated at 1, 5, 10, or 25 million cells/mL. This design was chosen for a number of reasons. First, the effect of varying concentration of tethered TGF*β* on cellular response was characterized, to provide insight into its bioactivity following the photoinitiated thiol-acrylate reaction used to form these PEG hydrogels. Secondly, this approach allowed us to test the bioavailability of the scaffold-tethered protein to cell binding and ultimately response. While this PEG scaffold is non-degradable, this approach provides confirmation that tethered TGF*β* is accessible to encapsulated cells. This observation is similar to the effects of adhesive peptides incorporated into non-degradable gels, where embedded cell can bind with the tethered ligand in the immediate pericellular space. Peptide signals often promote survival, but in a dose-dependent manner and often with a threshold. Thus, the amount of bioactive TGF*β* assayed should be a function of both TGF*β* concentration and cell density. Results are presented in Fig. 2.

For each tethered TGF*β* concentration, an increased luciferase activity was observed over the lowest cell seeding densities of 1, 5, and 10 million cells/mL. For gels formed with 12.5 nM TGF*β*, there was no statistically significant increase in luciferase activity when the cell seeding density was further increased from 10 to 25 million cells/mL. This result suggests that for the PEGDA hydrogels, at 10 MM cells/mL, tethered TGF*β* is the limiting factor for signaling, possibly due to an excess cell-receptor concentration (i.e., a maximum cell density) or through limitations in receptor access to the tethered growth factor. Increasing the concentration of tethered TGF*β* for higher cell densities would be expected to result in increased levels of luciferase activity. This hypothesis was confirmed for hydrogels containing 25- and 50-nM tethered TGF*β*. Luciferase activity was 7,800 AU for 25-nM hydrogels encapsulated at 10 MM cells/mL and increased to 12,600 AU at the higher 25-MM cells/mL seeding density. Likewise, 50-nM hydrogels had luciferase activity of 12,100 AU at 10 MM cells/mL, which increased to just over 21,800 AU at the higher density of 25 MM cells/mL. Interestingly, in both the 25- and 50-nM TGF*β* concentrations, increasing cell density by a factor of 2.5 resulted in a similar increase (165–180 %) in bioactive TGF*β*. These results with the PE25 cell line demonstrate the feasibility of presenting tethered TGF*β* as a bioactive signal to encapsulated cells; so we next sought to devise a study to test the ability of these materials to promote a more biologically relevant and complex response–chondrogenic differentiation of mesenchymal stem cells (MSCs).

### DNA quantification of hMSCs encapsulated in tethered TGF*β* hydrogels

**Fig. 3:**
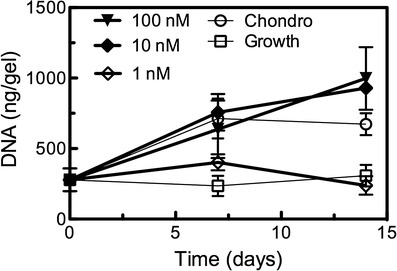
Viability of hMSCs encapsulated in tethered TGF*β* hydrogels. DNA content of cell-laden hydrogels was assayed over 14 days of culture, and used as a general correlative measure of cell viability. Hydrogels cultured in growth medium (used as a negative control for chondrogenesis) maintained initial cell counts, as did those incorporating 1-nM tethered TGF*β*. Chondrogenic media samples (positive control) exhibited an increase in DNA of approximately twofold, while TGF*β* tethered at 10 or 100 nM demonstrated similar increases in DNA content, suggesting high levels of viability of the encapsulated hMSCs. Results are presented as mean ± s.e.m. (*n* = 3)

After confirming that tethered TGF*β* maintained its bioactivity following photopolymerization, we then wanted to investigate whether such a platform could promote chondrogenic differentiation of encapsulated hMSCs. Cells were encapsulated at a density of 10 MM cells/mL in 10-wt% PEGDA hydrogels with 1 mM CRGDS peptide to promote integrin binding and survival. Immediately following photopolymerization, gels were assayed for DNA content via a Picogreen assay, and subsequently tested through culture for 14 days. Results are presented in Fig. 3. Cells cultured in growth medium showed little increase in DNA content over 14 days, agreeing with previous work on hMSC encapsulation [27]. In contrast, samples in chondrogenic medium exhibited a twofold increase in DNA content over the same time course. When tethered, TGF*β* was added to the hydrogel at the lowest concentration of 1 nM, DNA content also remained unchanged and approximated that of the profile of gels cultured in growth medium. At the higher concentrations of 10 and 100 nM TGF*β*, DNA content increased and was along the same order of magnitude as chondrogenic culture samples. These finding suggested that in all cases, cell survival following photoencapsulation was robust, a finding confirmed via histological and immunostaining analyses in following sections.

### GAG secretion of encapsulated hMSCs

**Fig. 4:**
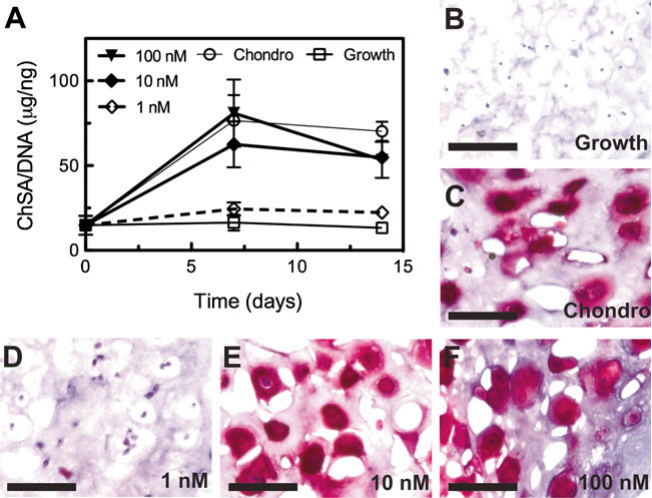
Glycosaminoglycan production by hMSCs encapsulated in tethered TGF*β* hydrogels. **a** ChSA production was quantified on a per gel basis via the DMMB assay, and is a measure of cellular GAG production, indicative of chondrogenesis. When TGF*β* was tethered at 1 nM, GAG production was limited and similar in magnitude to that of hydrogels cultured in growth medium, which acts as a negative control condition for chondrogenesis. At higher concentrations (10 and 100 nM) of tethered TGF*β*, GAG production exceeded that of the positive control (chondrogenic media), where TGF*β* was dosed solubly. **b**–**e** Safranin-O staining shows GAG distribution in the pericellular space for chondrogenic media culture (**c**), as well at 10 (**e**) and 100 nM (**f**) tethered TGF*β* gels. Gels culture in growth media (**b**) and those with 1-nM tethered TGF*β* (**d**) had negligible staining for GAGs. *Scale bar* = 100 μm

GAG production is one key earlier indicator of a chondrocyte-like phenotype [31–33]. Native cartilage is comprised primary of sulfated GAG chains and type II collagen. A DMMB assay was used to quantify secreted GAGs immobilized in the PEG hydrogel scaffold, as previously described [22, 23, 34]. Results are presented in Fig. 4a, with GAG secretion normalized to DNA (micrograms of chondroitin sulfate A (ChSA) per nanogram of DNA). For hydrogels cultured in growth medium, there was little to no measurable amount of GAGs. Day-0 content (for all samples) was 14.8 ± 5.6 μg/ng, and day-14 growth samples contained 13.2 ± 1.8 μg/ng. As a positive control, MSC-laden hydrogels cultured in chondrogenic medium had a marked increase in GAG production; at day 14, chondrogenic samples contained 70.2 ± 5.6 μg/ng. Interestingly, tethered TGF*β* at 1 nM provided a minimal increase over growth media, as cell-laden hydrogels were assayed to contain 22.4 ± 0.4 μg/ng at day 14. However, at the same time point, both 10 nM (54.9 ± 1.1 μg/ng) and 100 nM (53.4 ± 10.7 μg/ng) had GAG production similar to that of the positive control, where TGF*β* was delivered solubly. At day 14, each tethered TGF*β* gel condition resulted in a statistically significant amount of GAG production relative to that of the negative control (*p* < 0.01). Noteworthy, however, is the total amount of growth factor delivered to encapsulated cells in this study. In chondrogenic medium, TGF*β* is added to the media at a concentration of 5 ng/mL (0.2 nM), which is a much lower concentration than used in the hydrogels (1 to 100 nM). However, over 22 days of culture with media exchanges every 2 days, the total amount of TGF*β* delivered in the soluble form is 55 ng/gel. In contrast, incorporating tethered TGF*β* at 100 nM required 100 ng/gel, while the 10 nM gels used only 10-ng growth factor/gel to achieve a similar level of chondrogenesis. Further, tethering growth factors or other proteins into an implantable biomaterial provides a method to signal cells delivered in vivo, and this approach could be readily used to present multiple protein signals to cells, even in various regions of a single material.

Histological analyses of MSC cultured in tethered TGF*β* hydrogels for extended time periods provide a spatial representation of GAG deposition (Fig. 4b). Hydrogels were harvested at day 21, cryosectioned, and stained with Safranin-O, which stains GAGs red and counterstained with haematoxylin with stains nuclei blue/black. Gels cultured in growth medium had negligible red color surrounding nuclei, confirming data obtained from the DMMB assay. Likewise, gels incorporating 1-nM tethered TGF*β* have a low level of proteoglycan content, observed by faint staining. In contrast, hydrogels cultured in chondrogenic media had significant GAG deposition in the pericellular region and beyond, as did gels formed with either 10- or 100-nM tethered TGF*β*. Since this hydrogel platform has no degradable crosslinks, there is limited interstitial space for deposition of secreted ECM and this leads to some of the observed spatial heterogeneity. However, this data agrees with previously published work on GAG deposition for primary chondrocytes encapsulated in similar PEG-based materials [35, 36].

### Collagen II production in tethered TGF*β* hydrogels

**Fig. 5:**
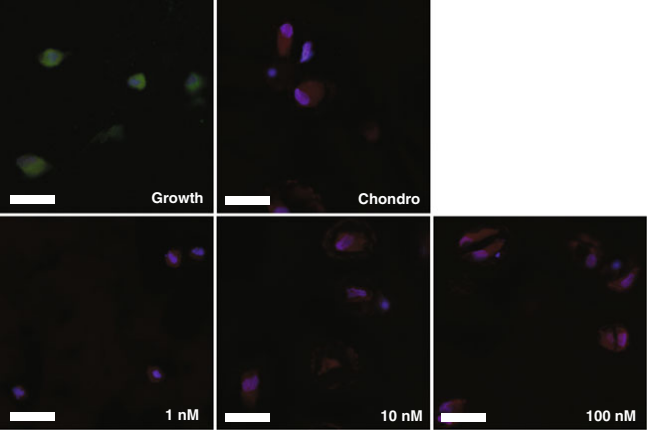
Immunostaining shows collagen II (*red*) or CD-105 (*green*) production by hMSCs encapsulated in PEG hydrogels. Nuclei are counterstained with DAPI (*blue*). Growth samples were positive for CD-105, indicating non-differentiated cells. Gels cultured in chondrogenic media with soluble TGF*β* expressed little CD-105, but were positive for collagen II, a hallmark of chondrogenic differentiation. Cells cultured in hydrogels with tethered TGF*β* at 1, 10, or 100 nM were all positive for collagen II and negative for CD-105. Scale bar = 200 μm

Collagen type II secretion is another hallmark of chondrogenic differentiation of hMSCs, while expression of the CD-105 cell surface marker is often used as one of the characteristic markers for undifferentiated hMSC [22, 33, 37]. To further characterize the role that tethered TGF*β* plays in promoting later stages of chondrogenesis, cell-laden gels were harvested after 21 days of culture for immunostaining. Samples were stained for type II collagen, CD-105, and DAPI to visualize cell nuclei. Representative images are presented in Fig. 5. Growth samples express the CD-105 marker to a greater degree than any samples cultured in chondrogenic conditions (green in Fig. 5), and only 2.2 ± 0.6 % were positive for collagen II. Interestingly, while 1-nM tethered TGF*β* hydrogels did not produce appreciable amounts of GAGs (Fig. 4), cells encapsulated in this gel formulation were negative for CD-105 staining, while 5.5 ± 0.7 % of cells counted were positive for collagen II, suggesting that the MSCs may be differentiating down other pathways. Cells encapsulated in hydrogels formed with either 10-nM (84.0 ± 13.9 %) or 100-nM (91.0 ± 0.2 %) tethered TGF*β* were largely positive for collagen II production (red in Fig. 5), similar to levels observed with the positive control (89.3 ± 7.2 %), chondrogenic medium with soluble TGF*β*.

## Conclusions

Thiolated TGF*β* was shown to maintain its bioactivity, and a surface ELISA method was developed to confirm that TGF*β* was detectable following covalent incorporation in PEGDA hydrogels. PE25 cells encapsulated in such tethered TGF*β* hydrogels were used to confirm that tethered TGF*β* maintained its bioactivity when presented in this fashion. When hMSCs were encapsulated in tethered TGF*β* hydrogels, the tethered growth factor promoted chondrogenic differentiation at levels equal to or exceeding that of positive controls, where TGF*β* was dosed solubly via culture medium. Additionally, tethered TGF*β* hydrogels utilized a lower total dosage to promote differentiation. Collectively, these results demonstrate the feasibility of delivering bioactive protein signals in a 3D culture platform to control stem cell fate, which may have further implications in the design of delivery vehicles for hMSCs to promote chondrogenesis and cartilage regeneration in vivo.
